# A Rare and Serious Unforeseen Complication of Cutting Balloon Angioplasty

**DOI:** 10.1155/2014/246784

**Published:** 2014-03-23

**Authors:** Praveen Vemula, Jagadeesh K. Kalavakunta, George S. Abela, Milind Karve

**Affiliations:** ^1^Department of Medicine, Division of Cardiology, Michigan State University College of Human Medicine, East Lansing, MI, USA; ^2^Division of Cardiology, Sparrow Health System, Lansing, MI, USA

## Abstract

Cutting balloon angioplasty (CBA) is one of the adept ways of treating “in-stent restenosis.” Various complications related to cutting balloon angioplasty have been reported including arterial rupture, delayed perforation and fracture of microsurgical blades. Here we report a very unusual and inadvertent extraction of a stent previously deployed in the ramus intermedius coronary branch by a cutting balloon catheter. This required repeat stenting of the same site for an underlying dissection. Even though stent extraction is a rare complication it can be serious due to dissection, perforation, and closure of the artery. Physicians performing coronary artery interventions would need to be aware of this rare and serious complication especially if any difficulty is encountered while withdrawing the cutting balloon. Therefore, after removal, cutting balloon should be examined thoroughly for possible stent dislodgment or extraction when used for “in-stent restenosis.”

## 1. Introduction 

In-stent restenosis is one of the important causes of failure of previously successful percutaneous intervention (PCI). Restenosis occurs secondary to neointimal tissue proliferation which is mediated by inflammation following arterial injury [1, 2]. Drug eluting stents (DES) have greatly reduced rates of restenosis compared to that of bare metal stents [3–5]. However, there are no clear guidelines for appropriate therapy of DES restenosis, probably due to low incidence of restenosis and its varied etiology [6]. Cutting balloon angioplasty (CBA) is one of the safest, cost effective, and adept ways of treating in-stent stenosis [7–9]. Cutting balloon has 3-4 microsurgical blades attached to its surface longitudinally. Inflation of the balloon will result in cutting of atherosclerotic plaque. This creates controlled injury and potentially reduces inflammation. Various complications related to CBA have been reported including arterial rupture, perforation, and fracture of microsurgical blades [10–12]. In this case report we present an unforeseen complication of CBA in which a previously deployed stent is inadvertently extracted along with the cutting balloon.

## 2. Case Presentation 

A 71-year-old Caucasian male patient with past medical history of coronary artery disease, hypertension, congestive heart failure, and stroke presented with the chief complaint of left arm pain and dyspnea. He had similar symptoms five months prior to the current admission at which time he underwent PCI with an Ion (Boston Scientific) DES stent (2.5 × 24 mm) placement to a moderate caliber ramus intermedius coronary artery with an 85% long segment stenosis. During this admission the patient presented with left arm pain which started while he was driving and was associated with dyspnea, both worsening with minimal exertion and relieved with sublingual nitroglycerine.

Cardiovascular examination revealed a grade II/VI midsystolic murmur best heard in the left midclavicular line in fourth intercostal space. No heaves, thrills, jugular venous distension, or carotid bruits were appreciated. Respiratory examination revealed fine crackles at the lung bases bilaterally. The rest of the physical examination was unremarkable. Chest X-ray demonstrated cardiomegaly with mild pulmonary vascular congestion. Electrocardiogram (ECG) demonstrated normal sinus rhythm with a right bundle branch block and an old anterior and inferior infarct. Laboratory data on admission demonstrated a troponin-I of 0.05 ng/mL (normal reference range < 0.02) and BNP > 5000 pg/mL, BUN of 20 mg/dL, and creatinine of 1.5 mg/dL.

**Figure 1:**
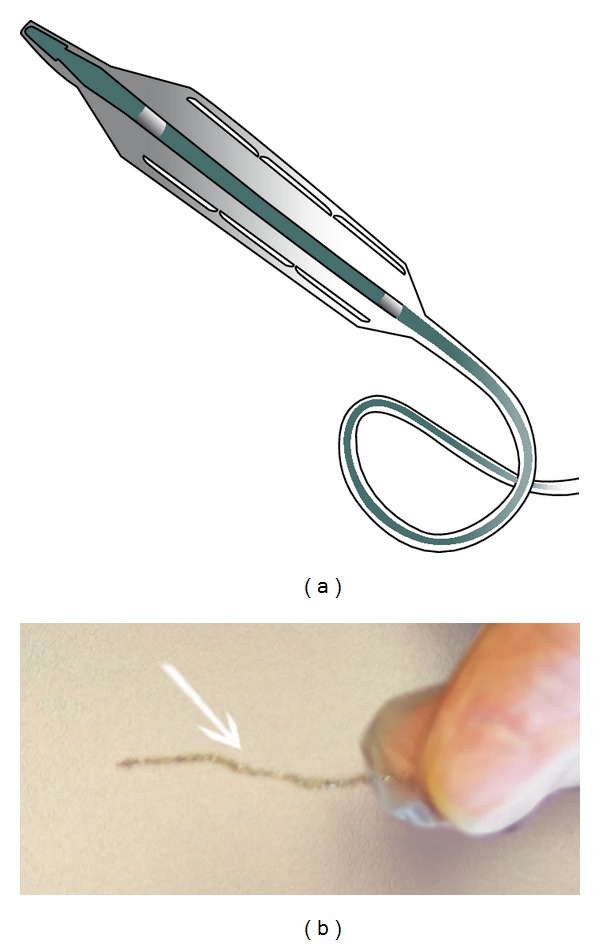
Flextome cutting balloon with extended blades (a). Extracted stent (b).

The patient was admitted to the cardiac step-down unit with the diagnosis of unstable angina. His cardiac biomarkers trended upward but were in an indeterminate range. Diagnostic cardiac catheterization was performed; coronary angiography demonstrated focal in-stent restenosis (70–80%) prior to a bend in the mid ramus intermedius artery. The decision was made to use CBA for the in-stent stenosis. With a 6 Fr Voda 3.5 guiding catheter (Boston Scientific) a 2.5 × 10 mm cutting balloon (*Flextome*, Boston Scientific) was introduced to site of “in-stent stenosis” over 0.014′′ BMW (balanced middle weight) guide wire and slowly inflated and deflated. Three inflations were performed (7, 7, and 10 atmospheres). However, while withdrawing the cutting balloon some difficulty was noted. Eventually the cutting balloon was slowly removed via the guiding catheter. The cutting balloon was carefully examined and we noticed a stent overlying the cutting balloon. It appeared that the previously deployed stent, 5 months earlier, had been pulled out along with cutting balloon (Figure 1).

**Figure 2:**
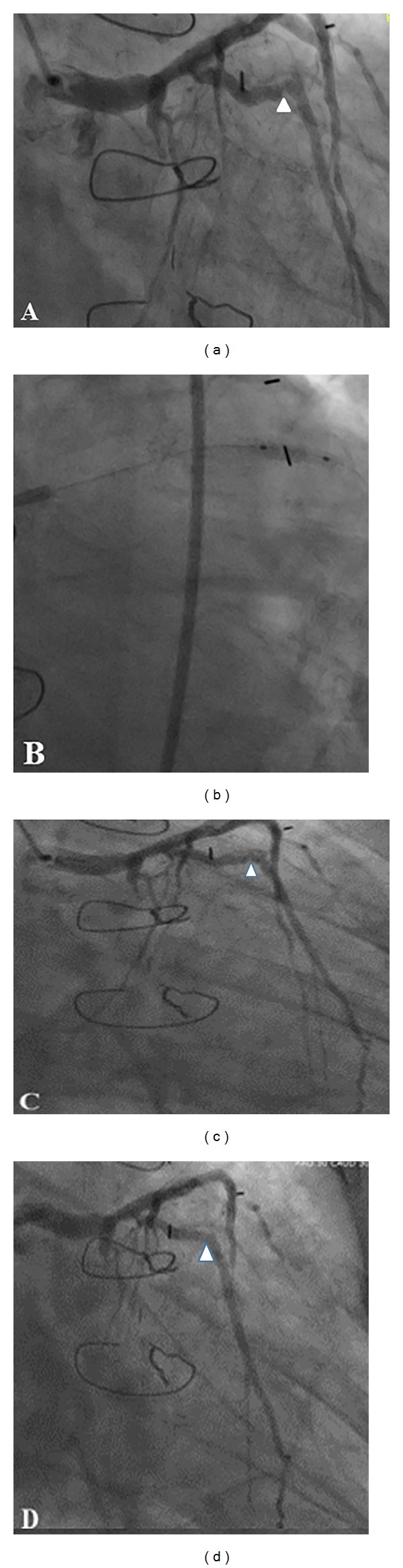
(a) RAO (right anterior oblique) caudal view of the left coronary artery system showing a significant lesion (arrow head) in the ramus intermedius branch at the bend in the proximal to mid portion. (b) Cutting balloon angioplasty (CBA) and post CBA angiogram (c) with dissection (arrow head). (d) Postintervention angiogram showed TIMI III flow without any dissection (TIMI: thrombolysis in myocardial infarction).

Repeat coronary angiography demonstrated a dissection at the previously stented site of the ramus branch. After several attempts a 0.014′′ whisper wire successfully crossed the arterial segment. Predilations were performed with 2.5 × 20 mini Trek compliant balloon and then 2.5 × 24 mm Ion (Boston Scientific) DES stent was deployed at 12 atmospheres. Poststent deployment angiogram was performed with and without the wire following the administration of intracoronary nitroglycerine. This showed 0% residual stenosis and TIMI III flow without dissection (Figure 2). Patient was returned to the floor in a stable condition and has remained asymptomatic on the follow-up clinic visits.

## 3. Discussion

Cutting balloon angioplasty is one of the common interventions for “in-stent restenosis.” It has practical benefits over percutaneous transluminal coronary angioplasty (PTCA). Cutting balloon has an advantage of better anchorage to slippery hyperplastic tissue over traditional PTCA balloons. Also the expanded blades of a cutting balloon can incise the tissue up to the stent cage. This provides an advantage of deep incision of hyperplastic tissue. Restenosis cutting balloon evaluation trial (RESCUT) clearly demonstrates these benefits of CBA over PTCA [13]. Other techniques (i.e., excimer laser atherectomy, mechanical atherectomy, radiation catheter, and rotational atherectomy) are also available to treat in-stent stenosis. These techniques are uncommon secondary to the cost involved in equipment and specialized training.

Since CBA is a widely used technique, it is important to understand various complications that may arise during and after this procedure. Various complications related to CBA have been reported including arterial rupture, delayed perforation, stent strut avulsion, and fracture of microsurgical blades [10–12]. However, very few cases have been reported regarding inadvertent stent extraction as a complication of CBA. In our case we report inadvertent extraction of ramus intermedius stent by an entrapped cutting balloon causing arterial dissection at previously stented site. Harb and Ling reported on the extraction of a stent deployed at the coronary ostium that was entrapped by the passage of a guide wire through protruding stent struts [14]. Similarly Almeda and Billhardt reported that a distal RCA stent was found attached to microtomes of a cutting balloon upon withdrawal [15]. Another case reported entrapment of stent secondary to blade fracture leading to stent extraction and acute occlusion of the coronary artery [16]. In our case the reason for entrapment is not clear. We carefully reviewed the previous stent angiograms which revealed appropriate size and deployment. Most likely the anatomical bend in the ramus intermedius artery as well as the entrapment of the cutting balloon blades in the stent struts maybe together caused the stent extraction. A repeat angiogram after the stent extraction suggested a dissection at the previously stented site requiring restenting of the ramus intermedius with a DES.

## 4. Conclusion

To our knowledge stent extraction is a very rare complication of CBA. In our case the inadvertent stent extraction by a cutting balloon also resulted in arterial dissection. Although it is a rare complication, stent extraction can cause a serious coronary artery injury. Physicians performing coronary artery interventions should be aware of this rare and serious complication especially if they encounter difficulty while withdrawing the cutting balloon. After removal, cutting balloons should be inspected thoroughly for an extracted stent.
